# Renal Urologic Anomalies Presenting In Adult Identical Twins

**DOI:** 10.1100/tsw.2004.56

**Published:** 2004-06-07

**Authors:** Robert A. Edelstein, Richard E. Altman, Peter Koch-Weser

**Affiliations:** Department of Urology, Lowell General Hospital, Lowell, Massachusetts, USA

## Abstract

Two sets of identical adult twins recently presented to our hospital. In one case, the patients demonstrated (ipsilateral) renal agenesis. In the other, the patients presented approximately one year apart with symptomatic (ipsilateral) ureteropelvic junction obstructions. Although the literature suggests a few reports of this type in the pediatric and newborn population, the authors are unaware of similar reports in adults.

## CASE REPORTS

Case 1: D.K., a 35 year old female presented to our hospital at 29 weeks of gestation during an otherwise unremarkable pregnancy with a complaint of right flank discomfort. Renal ultrasound demonstrated marked right unilateral hydronephrosis, but did not elucidate the site of obstruction. A limited "one shot" intravenous pyelogram (IVP) was performed, demonstrating a typical appearance of ureteropelvic junction (UPJ) obstruction (Figure 1). A complete history revealed that one year earlier, her identical twin sister (M.B.) had also presented as an out patient to our facility with similar complaints. Her evaluation revealed a diagnosis of ipsilateral UPJ obstruction (Figure 2). She underwent an uncomplicated dismembered pyeloplasty, revealing evidence of a stenotic segment at the UPJ as the cause of the obstruction. No evidence of a crossing lower pole vessel was identified. At this time, D.K. has not undergone surgical intervention.

Case 2: A 43 year old woman presented to her primary care physician complaining of back pain. An IVP was performed, demonstrating a normal left kidney and absence of the right kidney (Figure 3). There was no past history of urologic disease or surgery. When informed of the result, the woman stated that she had not been previously aware that she only had one kidney, and wondered whether her identical twin sister might be similarly affected. One week later, the twin sister was evaluated with an IVP, demonstrating unilateral absence of the right kidney, and a solitary left kidney almost identical in appearance to that of her sibling (figure 4).

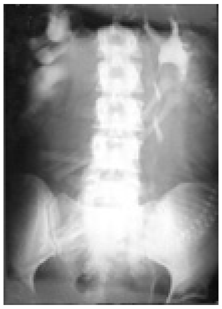

**FIGURE 1**. Excretory phase of intravenous "one shot" pyelography demonstrating appearance of obstructed renal pelvis. Note intrauterine fetal bony elements representing third trimester pregnancy.

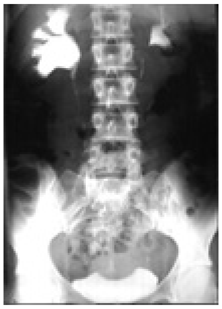

**FIGURE 2**. Excretory phase of intravenous pyelography demonstrating obstruction at the ureteropelvic junction. This patient is the identical twin of the patient shown in Figure 1.

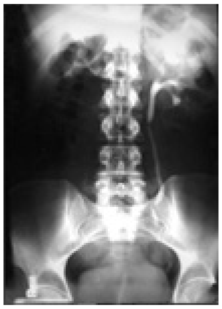

**FIGURE 3.** Index patient, demonstrating unilateral absence of right kidney.

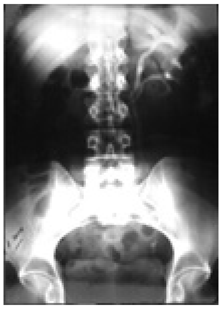

**FIGURE 4.** Identical twin sister of patient shown in Figure 3, demonstrating ipsilateral absence of right kidney. Note partial duplication of ureter.

## DISCUSSION

Many reports in the literature discussing congenital urologic pathology in twins focus on the pediatric population. In a summary by McCandless et. al. (1) urethral valves and reflux accounted for the majority of cases. Very few reports document the occurrence of ureteropelvic junction (UPJ) obstruction (1,2). UPJ obstruction is almost always congenital in origin. A variety of problems are thought to be causative, including such extrinsic factors as crossing lower pole renal vasculature, adhesive bands, insertion of the ureter into a high position on the renal pelvis (preventing dependent drainage), as well as intrinsic ureteral problems such as deficient musculature or mucosal folds. In the era of pre-natal ultrasonography, many cases of UPJ obstruction are detected antenatally, thus allowing for the consideration of early intervention. In practice, however, an adult will present from time to time with a previously undiagnosed UPJ obstruction. In some cases, a careful history may suggest some of the typical historical features, such as pain with diuresis (e.g. after consumption of alcohol or caffeine) or a history of febrile urinary tract infections, but this is not always the case. Following the initial diagnosis, most patients will undergo some type of renal functional and anatomic study, allowing decisions to be made on the type of intervention (nephrectomy versus reconstruction). In the past decade, the development of many types of "minimally invasive" antegrade and retrograde endopyelotomy techniques have been developed, allowing selected patients to avoid the traditional open pyeloplasty. Unilateral renal agenesis in the absence of other anomalies is not particularly rare, but reports of the incidence of this condition in twins is lacking. Most affected individuals with a normal solitary renal unit will have no functional limitations, and therefore may not be detected unless they undergo body imaging (often for some other reason). A paucity of cases in the literature focus on the relatives of index patients presenting with bilateral renal agenesis, and of these, only a few were noted to have siblings with unilateral agenesis (summarized in Carter et. al (3). Of the cases of ("probably monozygotic") twins reported in this large survey, only one sibling of an index patient had a form of unilateral renal agenesis (3). These two adult cases point out that similar developmental anomalies may develop in twins. Based on these experiences, we believe that the identical twin siblings of adult individuals presenting with (likely) congenital urinary tract abnormalities should be offered screening. This has been suggested in the pediatric population (2). As ultrasonography provides a non-invasive, risk free assessment of the number and architecture of the renal units, we suggest that this should be the imaging study of choice.
